# Supplementary material for: Longitudinal ultrasonic dimensions and parametric solid models of the gravid uterus and cervix
Source: PLoS One. 2021 Jan 28;16(1):e0242118. doi: 10.1371/journal.pone.0242118 (PMC7842891; doi:10.1371/journal.pone.0242118)
Supplement: S1 Appendix — (PDF) [file pone.0242118.s001.pdf]

S1 Appendix. Parametric Solid Model Solidworks Workflow To build the *Default Configuration*, first the sagittal intrauterine wall was defined in the front-plane. The anterior intrauterine wall was built using two equal quarter ellipses, where the inferior-superior radial value was half of the inferior-superior intrauterine diameter (UD1) and the anterior-posterior radial value was the perpendicular distance from the midpoint of UD1 to the anterior intrauterine wall (UD2) (Fig. 5a). The posterior intrauterine wall was built using either a single spline or two quarter ellipses and a spline. UD3a, UD3, and UD3b were used to define the posterior side in both cases, with UD3a placed superiorly to UD3b (Fig. 5a). In the case of the single spline, the ends of the spline were defined to be tangent where it connects to the quarter ellipses of the anterior side. For the two quarter ellipses and a spline as the posterior side, UD3a and UD3b were used as the anterior-posterior radial values, and UD3 was a connecting point for the spline between them. The spline was defined to be tangent to the quarter ellipses at the shared points of UD3a and UD3b.

Next, the outer uterine wall was defined in the front-plane. The build process from the intrauterine wall was repeated, but the sagittal uterine thicknesses were added. Thus, UT1 was added superiorly to the inferior-superior intrauterine diameter (UD1) to define the superior outer uterine wall, UT2 was added anteriorly to UD2 for the anterior outer uterine wall, UT4 was added inferiorly to UD1 for the inferior uterine wall, and UT2 was added posteriorly to UD3a, UD3, and UD3b for the posterior uterine wall, as no posterior uterine wall thickness was collected (Fig. 5b).

To construct the three-dimensional uterus, a *Lofted Boss/Base* function was used. In order to execute a *Lofted Boss/Base* function, *Profiles* and a *Guide Curve* were defined.

Small ellipses were used as the *Profiles* to the right and left of the sagittal plane. The ellipse size was defined using UD2/500 as the vertical radius and half of UD1/500 as the horizontal radius (Fig. 5c). This was done to ensure the guides were similar in overall shape to the sagittal uterine wall, but small enough to not affect future finite element analysis. With respect to the sagittal plane, the ellipses were placed at the midpoint of UD1 and the midpoint of UD2 and UD3, and were placed a distance of half of UD4 plus UT3 from the front-plane to define the right and left-most walls of the uterus. The *Guide Curve* sketch plane was defined by the superior-most points of the elliptical left and right profiles and the sagittal uterine wall profile. The *Guide Curve* was then drawn as a half-ellipse connecting the superior points of the elliptical left and right profiles and the sagittal uterine wall profile (Fig. 5c).

Next, cervical placement was determined in the front-plane by finding the intersection point of a guide line posteriorly parallel to the inferior-superior intrauterine diameter (UD1) and the inferior side of the sagittal intrauterine wall at a distance of the posterior cervical offset (PCO) (Fig. 5d). From this point, a plane was defined to be perpendicular to the inferior intrauterine wall. A second plane was placed at an angle of AUCA to the perpendicular plane (Fig. 5d). A circle was sketched on this plane with a diameter of CD1 and extruded a length of CL+IS using the *Extruded Boss/Base* feature, creating the cervical geometry (Fig. 5e). The intrauterine cavity was then generated using the *Lofted Cut* function (Fig. 5f). Having similar requirements as the *Lofted Boss/Base* function, the *End Guides* were the same ellipses, but rather placed at a distance of half of UD4 from the Front Plane on its left and right sides. The *Guide Curve* was defined in a similar fashion to the outer uterus loft *Guide Curve*. On the same plane as the definition of the gross cervical geometry, a circle with a diameter of CD2 was sketched and an *Extruded Cut* was performed at a length of CL+IS (Fig. 5f). Finally, fillets were added to the internal os, external os, exocervix, and outer uterocervical junction (Fig. 5g). With the completion of the *Default Configuration*, a design table was enabled and all patient-specific ultrasound measurements were input. Patient-specific parametric models were then automatically generated.
