# Supplementary material for: Longitudinal ultrasonic dimensions and parametric solid models of the gravid uterus and cervix
Source: PLoS One. 2021 Jan 28;16(1):e0242118. doi: 10.1371/journal.pone.0242118 (PMC7842891; doi:10.1371/journal.pone.0242118)
Supplement: S2 Appendix — (PDF) [file pone.0242118.s002.pdf]

S2 Appendix. Parametric Model Validation Method The commercial software Materialise Mimics (Research 20.0, Materialise MV, Leuven, Belgium) was used to segment the MRI image stacks for 8 term pregnant women prior to caesarean delivery [4]. On average, every third image was selected for manual segmentation using the paint tool. An interpolation was performed among segmented images to create a complete segmentation. A three-dimensional geometry was then created by exporting the resulting segmentation as a stereolithography (STL) formatted surface. The STL file was then conservatively smoothed using a commercial software 3D-Coat (Pilgray, Kiev, Ukraine) to preserve the overall geometric features and eliminate minor surface imperfections. Of the 8 STL files, 5 were used for parametric model validation, as 3 geometries had large gaps in the uterine wall. These 5 STL models are available through the Columbia University Library's permanent Academic Commons collection (url: https://doi.org/10.7916/d8-gxv7-2z02).

Segmented MRI-derived solid models were measured using the 2D ultrasound measurement protocol instead of MRI images. Each STL volume was then imported into Solidworks 2018-2019 (Dassault Systemes, Vélizy-Villacoublay, France) as a solid body. The TA sagittal plane was chosen as the section with the largest cervical canal width, and the TA axial plane as the one with the largest internal diameter. From these planes, all dimensions were taken using the 2D ultrasound measurement protocol described above.

Using the measurements taken, a parametric solid model was built from each MRI-derived solid model in order to compare the complex MRI-based model to the simplified ultrasound-based model. The similarity of the parametric models to the MRI-derived solid models was analyzed by comparing their shapes in the sagittal plane. Sagittal slices of models were aligned so the inferior-superior intrauterine diameters (UD1) were colinear. The models were also aligned using CL in the sagittal plane to compare uterocervical junction shape. The volume of the MRI-derived and parametric models was also compared. This was accomplished by using the "Mass Properties" tool in Solidworks. The volume percent error (Error) between the models was computed by finding the volume difference between the MRI-based model volume (MRI) and parametric model volume (Para), then dividing by the MRI-based model volume and multiplying by 100 (Eq. 3).

$$\text{Error} = \frac{MRI - Para}{MRI} * 100, \tag{3}$$

Validation against MRI-Derived Models The shape of the parametric and MRI-based models is compared by overlaying in the sagittal plane. The overlays are shown in S2 Fig. 1. The parametric model is also compared to the MRI-based models through volume measurements. The average error in volume between the MRI-derived solid models and parametric models is $7 \pm 7\%$. For MRI patients 1, 2, and 5, the parametric model underestimates the volume of the MRI-based model. The parametric model overestimates the volume of the MRI-based model for patients 3 and 4.

S2 Fig. 1 Comparison of Solidworks sagittal shape to MRI-based model sagittal shape Solidworks model sagittal slices overlaid on MRI-based model sagittal slices. Top row aligned using the inferior-superior intrauterine diameter (UD1), bottom row aligned using the cervical length (CL).

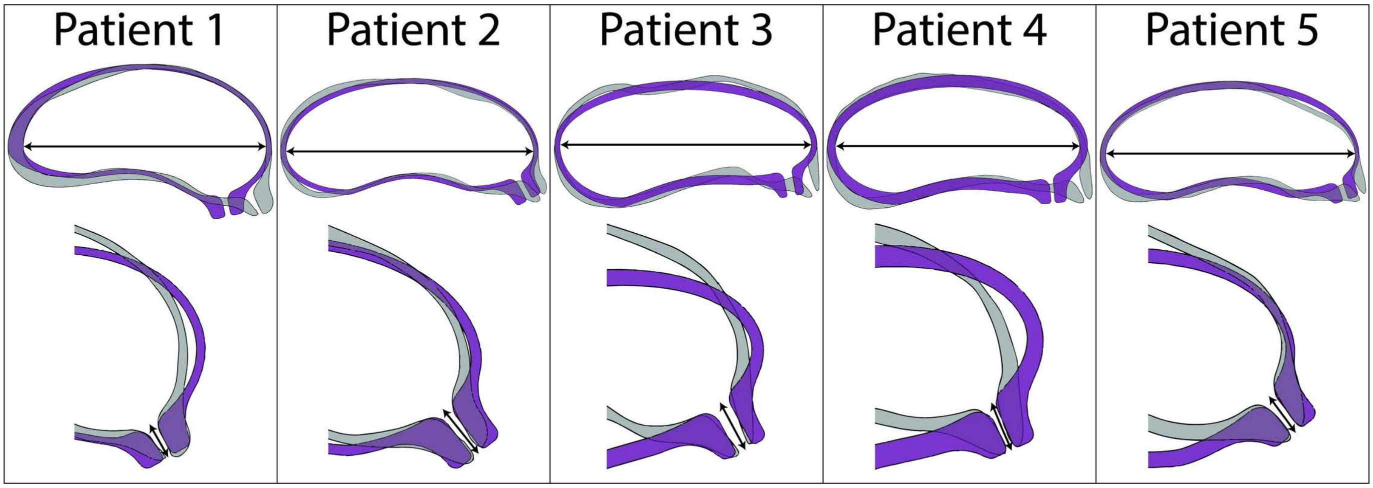

By visual comparison, the sagittal profiles are a good visual match for patients 1, 2, and 5. The visual match in sagittal profile shape is fair for patient 4, and patient 3 did not match well. The MRI-based solid models which had the best visual match with their associated parametric model exhibited features in accordance with assumptions made during the parametric model build, such as having a maximum anterior radial diameter close to the center of the inferior-superior axis, and local extrema of the posterior uterine wall equally spaced along it. However, the uterocervical junction shape is not well captured in the parametric models with exception in patient 3, where a similarity in shape is observed. The uterocervical junction must be accurately captured to utilize this modeling process in future computational studies. This area likely has consequential tissue stretch and stress and plays a fundamental role in late pregnancy and birthing dynamics. One possible alteration to better capture the uterocervical junction is to redefine UD3a and UD3b as local extrema of the posterior wall on the superior and inferior halves of the inferior-superior intrauterine diameter (UD1), respectively, and additional measurements taken as locator dimensions along UD1. The results of redefining UD3a and UD3b as such are shown in S2 Fig. 2. Incorporating these measurement definitions into the parametric models results in much better agreement of uterocervical junction shape between the MRI-based model and parametric model for all patients, as well as match in overall sagittal profile shape. This methodology has limited use as not all posterior walls have clear extrema, in which case the equidistant placement of posterior intrauterine wall diameters is better. The equidistant measurement definition will likely offer better results in early gestation, before the uterus has abutted against the spine. Certain geometric features should offer insights into which measurement definitions would more accurately model individual patients. Further studies are required as not all geometries can be represented using the presented processes.

S2 Fig. 2 Comparison of Solidworks sagittal shape to MRI-based model sagittal shape for alternate measurement method. Solidworks model with alternate measurement method sagittal slices overlaid on MRI-based model sagittal slices. Top row aligned using the inferior-superior intrauterine diameter (UD1), bottom row aligned using the cervical length (CL).

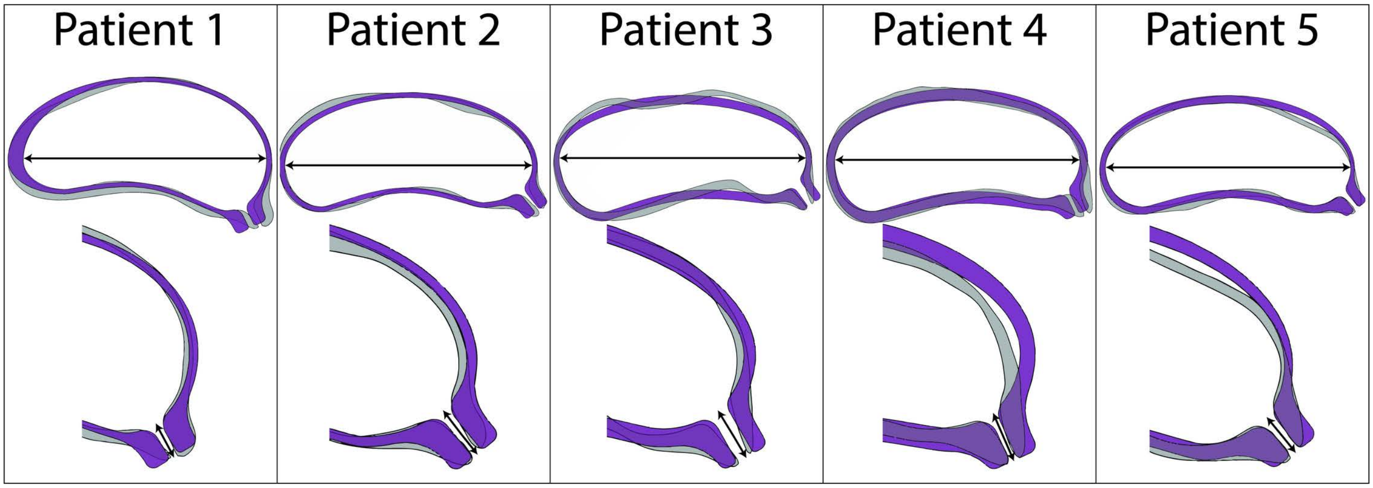

The coronal and axial planes are not well matched between the MRI-based models and parametric models. This is due to the use of ellipses to model the coronal and axial shape, with the largest transverse diameter assumed to be at the midpoint of the inferior-superior intrauterine diameter (UD1). The MRI-based models show the coronal shape of the uterus in late gestation has a maximum transverse diameter occurring at approximately 60% of UD1 from the inferior uterine wall. Therefore, for late gestation models, it is not appropriate to use an ellipse as the coronal shape of the uterus and the parametric construction method must be revised to account for the tapering of the transverse uterine diameter towards the uterocervical junction, as observed in the MRI-based models. It is currently unknown how the uterine shape in the coronal and axial planes change throughout pregnancy, and additional ultrasound images are necessary to characterize the evolution of the inferior portion of the uterus throughout pregnancy. A better understanding of the shape evolution of the uterocervical junction would be of great impact in future computational studies.
