# Supplementary material for: Longitudinal ultrasonic dimensions and parametric solid models of the gravid uterus and cervix
Source: PLoS One. 2021 Jan 28;16(1):e0242118. doi: 10.1371/journal.pone.0242118 (PMC7842891; doi:10.1371/journal.pone.0242118)

S3 Appendix. Patient 2D ultrasound dimension measurements with gestational in the supine position averaged across sonographers

S3 Fig. 1 Uterine Diameters Uterine diameters with gestational age in the supine position averaged across sonographers. Shown are inferior-superior intrauterine diameter (UD1), perpendicular distance between the midpoint of UD1 and the anterior intrauterine wall (UD2), perpendicular distance between the midpoint of UD1 and the posterior intrauterine wall (UD3), perpendicular distance between 25% and 75% of UD1 from the superior to the posterior intrauterine wall (UD3a & UD3b), and the left-right intrauterine diameter (UD4).

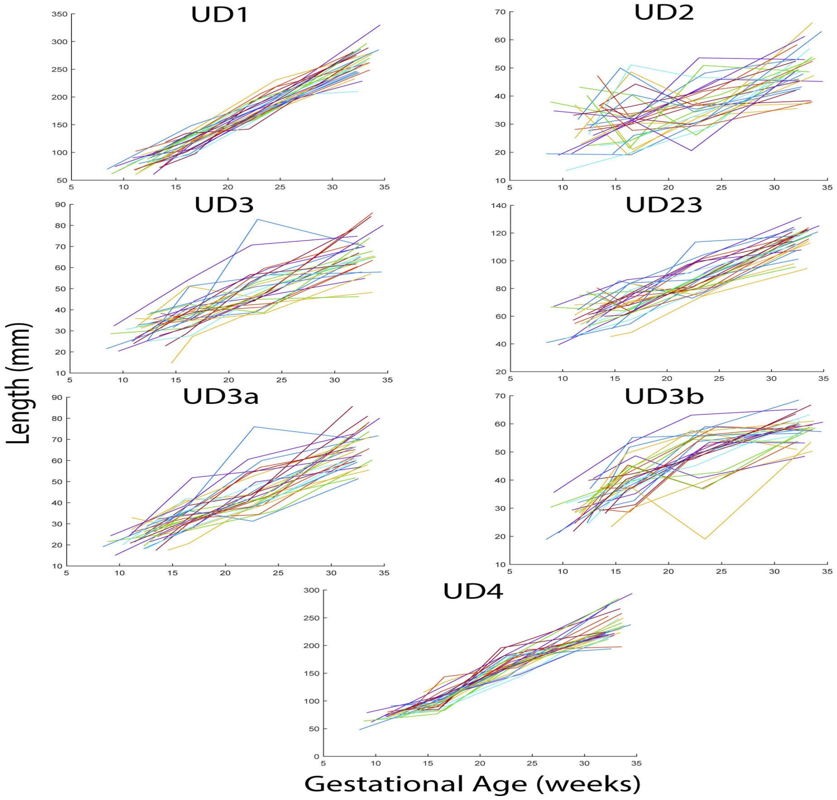

S3 Fig. 2 Uterine Wall Thicknesses Uterine wall thicknesses with gestational age in the supine position averaged across sonographers. Shown are fundal uterine wall thickness (UT1), anterior uterine wall thickness (UT2), left or right uterine wall thickness (UT3), and the lower uterine segment thickness (UT4).

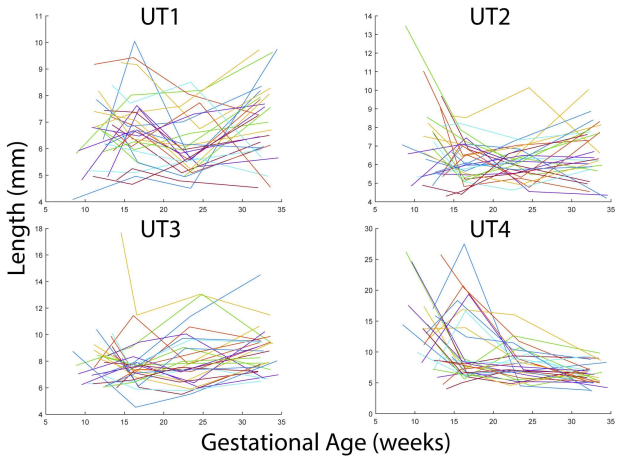

S3 Fig. 3 Cervical Measurements Cervical measurements with gestational age in the supine position averaged across sonographers. Shown are the posterior cervical offset (PCO), anterior uterocervical angle (AUCA), cervical length (CL), isthmus length (IS), outer diameter of the cervix (CD1), and the diameter of the inner cervical canal diameter (CD2).

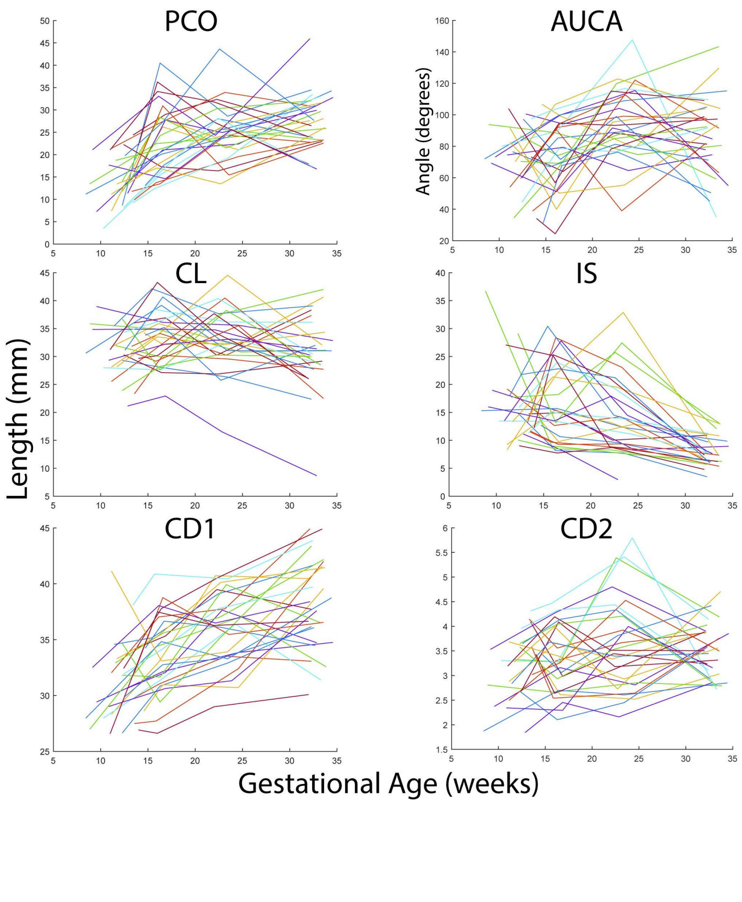

Supplement: S3 Appendix — (PDF) [file pone.0242118.s003.pdf]
